# Bicultural identity among economical migrants from three south European countries living in Switzerland. Adaptation and validation of a new psychometric instrument

**DOI:** 10.1186/1471-244X-7-17

**Published:** 2007-05-09

**Authors:** Ariel Eytan, Nuria Jene-Petschen, Marianne Gex-Fabry

**Affiliations:** 1 Department of Psychiatry, Adult Psychiatric Service, Geneva University Hospitals, Switzerland

## Abstract

**Background:**

Acculturation is one of the determinants of mental health among immigrants. Evaluating adaptation to the host culture is insufficient, since immigrants will develop various degrees of bi- or multicultural identity. However, mental health professionals lack simple and easy to use instruments to guide them with bicultural identity evaluation in their practice. Our aim was to develop such an instrument to be used for clinical purposes among economical migrants from three South European countries living in Geneva, Switzerland.

**Methods:**

We adapted from existing instruments a 24 item bi-dimensional scale to assess involvement in both culture of origin and host culture. The study included 93 immigrant adults from three south European countries (Italy, Portugal and Spain). Thirty-eight patients were recruited in an outpatient treatment program for alcohol-related problems and 55 participants were hospital employees.

**Results:**

The questionnaire was rated as easy or rather easy by 97.8% of participants. Median time to complete it was 5 minutes. The instrument allowed discriminating between patients and healthy subjects, with scores for Swiss culture significantly higher among hospital workers. The subscales related to culture of origin and host culture displayed adequate internal consistency (Cronbach's alpha 0.77 and 0.73 respectively).

**Conclusion:**

It is possible to assist clinicians' assessment of cultural identity of Italian, Portuguese and Spanish economical immigrants in Switzerland with a single and easy to use instrument.

## Background

Various definitions of culture and cultural identity exist. In the psychological and medical literature, culture is usually considered as being made of the schemes and values, explicit and implicit, symbolically transmitted from one generation to the next that determines some of the human group behaviors. Culture is a social construct implying highly variable systems of meanings.

When individuals migrate from one country to another, it is likely that their cultural and ethnic identity will change [1]. When cultural groups come into contact, transfer of schemes and values occurs in both directions [2]. This conception of reciprocal influences on cultural identities, leading to some degree of bicultural identity, is quite recent. Indeed, it emerged in the nineteen seventies with the study of various immigrant communities in the United States. It differs from an older position that viewed cultural adaptation as a unidirectional, acculturative process whereby immigrants change their behavior and attitudes toward those of the host society. This unidirectional view was partly inherited from culturally imperialist times when cultural changes were understood as "the modification of a primitive culture by contact with an advanced culture" (American Heritage Dictionary, 1978, quoted by Escobar and Vega) [2]. With the process of globalization, it can be expected that most people around the world will develop a different form of bicultural identity, combining their local identity with an identity linked to the global culture [3].

These cultural and ethnic identity variations related to migration, either forced or voluntary, may impact upon individuals' mental health and both psychiatric and general medical services utilization. Cultural differences in definitions of what constitutes normal and abnormal behavior, as well as differences in explanations and presentation of mental illness are examples of the factors involved [4]. Therefore it is important to be able to assess and measure cultural identity in the context of mental and general health care in various migrant groups. Several acculturation inventories and scales were developed for this purpose since the sixties, mainly for use among Latin American immigrants in the United States. Initially based on the "mutual exclusion" model of acculturative change mentioned above, these instruments were mono-directional, contrasting Hispanicism versus Americanism [5]. Traditionally also, instruments used to address different facets of language behavior as the core dimension of acculturation. Other components of cultural identity were added over time, such as spirituality, cultural values and everyday life indicators of cultural identity like cooking or reading preferences.

With the emergence of the concept of bicultural identity, instruments were developed for assessing involvement in the cultures of both the society of origin and host society. Several authors have shown that the bi-dimensional model is a more valid and useful operationalization of acculturation than mono-dimensional models [6]. For example, a recent instrument was designed to measure biculturality among Mexican adolescents in the United States [7]. Among these various instruments, scales, questionnaires or inventories, some are short and simple to administer, others are more exhaustive or require specific training [8].

In the contemporary world, characterized by an increase of migration movements and cultural diversity, mental health professionals have to deal with patients from diverse cultural backgrounds. It thus becomes insufficient to have one ethnically and culturally specific instrument or bicultural scale, whatever its intrinsic qualities. We suggest that it is possible to assess bicultural identity across national groups, especially if those groups share similar migration pathways and motivations.

These were the reasons for designing a multi-dimensional (including language and other cultural characteristics), bi-level (individual and social group), bi-directional (country of origin and host country) and multiethnic (Italian, Spanish and Portuguese) scale (Table 1). The main goal for developing this instrument was to help with services and treatment, but the scale could also be valuable for epidemiological studies. The specific objective of the present study was to assess the acceptability of such a new brief scale and provide support for its validity.

**Table 1:** Scales for measuring cultural identity: from the origins to a contemporary view

Mono-dimensional (e.g. language behaviors)	→	Multi-dimensional (e.g. values, spirituality)
Mono-level (the individual)	→	Bi-level (the individual and the social group)
Mono-directional (acculturation to the host country)	→	Bi-directional (bicultural identity)
Mono-ethnic (designed for one group of migrants)	→	Multi-ethnic (applicable across several ethnic or cultural groups)

## Methods

### Local background

Switzerland has been a country of economical immigration since the end of the 19th century. Between 1974 and 1979, following the energy crisis, there was a marked decrease of the number of foreign residents. Since 1980, there is a renewed demand for foreign laborers, fluctuating according to the economic situation. Among the most represented nationalities, South European citizens (from Italy, Spain and Portugal) share some cultural characteristics, e.g. value of work, family support and social integration.

However, there are some differences in the immigration patterns and chronology. Whereas Spanish immigration has been largely stable over the years, the majority of Italians arrived in Switzerland between 1960 and 1974, while Portuguese immigration is more recent, with a peak between 1980 and 2000. Thus, organization levels of the respective foreign communities in Switzerland do vary. So do family issues, for example the transmission of culture of origin to the second or even third generation. Italian, Spanish and Portuguese being Latin languages, linguistic adaptation in a French speaking environment like Geneva is usually not an insuperable difficulty.

Overall, South European economical immigrants belong twice more often to the "working poor" category than Swiss workers, at least partly due to lower qualification and education levels. The former are also significantly more at risk of being unemployed and requiring social welfare than the latter. Although illegal immigration is a problem in Switzerland, it does not significantly affect South European populations, but rather Eastern European, African and South American immigrants [9].

### Development of the questionnaire

We first examined existing bicultural scales, especially those designed for Latin American immigrants in the US, but also those validated among Asian immigrants in the US [10,11] and in Australia [12]. Based on them, and principally the one constructed by Cortés *et al*. [13], an initial set of items that appeared clinically relevant in our multicultural context was selected and translated into French (the official language in Geneva). Our clinical experience with migrants in the Swiss community led to the subsequent adaptation of these items and the inclusion of two additional ones (feeling at ease for expressing emotions in French versus in the individual's mother tongue; importance for the person to rest, after his or her death, in Switzerland versus in country of origin). Indeed, these two topics are often raised by patients in clinical practice. A 24-item Likert scale was thus obtained that considered 12 items for the culture of origin and 12 items for the Swiss culture. It is short, single-page and can be used as a self-report, possibly with the assistance of a clinician. Items are aimed at grasping everyday life, easily identifiable indicators of the individual's cultural identity. The English equivalents (partially reproduced from the above mentioned original instrument by Cortés) are presented in this study. The French original version is available upon request.

Each item was rated on a 4-point scale, anchored at 1 = Not at all, 2 = A little, 3 = Quite a bit and 4 = Very much. Total scores for the culture of origin and the host culture were obtained by summing the 12 items of each subscale. Theoretical variability was thus between 12 and 48 for both scores.

### Subjects

The study sample was selected among Portuguese, Spanish and Italian first-generation economical migrants settled in the Geneva community. It consisted in two sub-samples of adults. The first sub-sample included 42 patients recruited at an outpatient treatment facility for alcohol-related disorders. One of the authors (NJP), a trained psychiatrist, proposed the study to all eligible patients in her practice over a one month period. The second sub-sample included 55 active hospital professionals recruited among health care and administrative staff through an advertisement in the news bulletin, mailed to all hospital employees. Volunteers were invited to call the clinician in charge of the study (NJP). She made appointments with the participants in the most convenient place for them, usually their working place at the hospital. Both patients and professionals answered the questionnaire with her assistance. All participants had a sufficient knowledge of the French language to understand the questions and answer them. The ethics committee of the Psychiatry Department, Geneva University Hospitals, approved the study protocol. All subjects provided written informed consent before entering the study. No financial compensation was given.

### Statistics

*Acceptability *was measured through the percent of fully completed questionnaires and questions to the subject and reference psychiatrist about how easy it was to complete it. The time needed to fill the questionnaire was also recorded.

*Subscale score distributions *were assessed through descriptive statistics. The percent of the sample achieving the lowest and highest possible scores were calculated (floor and ceiling effects).

*Internal consistency reliability *of each subscale was estimated with the Cronbach's alpha coefficient. *Item internal consistency *was assessed through the correlation coefficients of each item score with the total subscale score calculated after excluding the tested item.

The *factorial structure *of the complete 24-item scale was investigated with principal components analysis, with varimax rotation. The number of factors was determined according to the scree plot method, i.e. retaining the principal components corresponding to the steep part of the graph.

*Construct validity *was examined by testing the following hypotheses. Firstly, it was assumed that patients would display lower scores for host culture than hospital professionals (*discriminant validity*). Secondly, in the absence of a validated reference instrument, *convergent validity *was approached through the hypothesis that scores for origin and host cultures would respectively decrease and increase with longer stay in Switzerland. Similar associations were expected with younger age at arrival in Switzerland. Thirdly, it was assumed that subjects who more frequently return to their country of origin or keep more frequent contact with relatives or friends abroad would score higher on the subscale relative to their culture of origin, but not on the one relative to the Swiss culture.

Group comparisons were performed with the Fisher's exact test for categorical variables, the Mann-Whitney U test and Kruskal-Wallis one-way analysis of variance for ordinal scores. The Wilcoxon signed ranks test was used for within subject comparisons. Associations were tested with the Spearman rank order correlation coefficient. Significance level was set at 0.05 (two-sided tests). Statistical analysis was performed with SPSS version 11 (SPSS Inc., Chicago, IL).

## Results

### Sample characteristics

Of the initial sample of 97 subjects (55 hospital workers and 42 patients), four were excluded because of incomplete questionnaires (n = 3) or incomplete socio-demographic data (n = 1). Statistical analysis thus concentrated on 93 subjects (55 hospital workers and 38 patients), whose characteristics are presented in Table 2. Compared with hospital workers, patients were more often male (78.9% vs 49.1%, P = 0.005), less often married (57.9% vs 78.2%, P = 0.042) and, as expected, less frequently professionally active (42.1% vs 94.5%, P < 0.001). The two samples also differed with respect to their country of origin, with a majority of patients from Portugal and most hospital workers from Spain (P = 0.026). No significant difference was observed for age (median values 45 and 44 for patients and hospital workers, respectively), length of stay in Switzerland (23 vs 21 years) or age at arrival in Switzerland (24 vs 23 years).

**Table 2:** Sample characteristics

Characteristic	Hospital staff (n = 55)		Patients (n = 38)		P^a^
Male sex (n; %)	27	49.1	30	78.9	0.005
Age (median; min – max)	44	19 – 68	45	28 – 74	0.745
Primary school only (n; %)	27	49.1	24	63.2	0.208
Lives as a couple, with or without children (n; %)	45	81.8	25	65.8	0.092
Professionally active (n; %)	52	94.5	16	42.1	<0.001
Country of origin (n; %)					0.026
Spain	31	56.4	13	34.2	
Portugal	14	25.5	20	52.6	
Italy	10	18.2	5	13.2	
Age at arrival in Switzerland (median; min – max)	23	9 – 41	24	1 – 40	0.748
Length of stay in Switzerland, yrs (median; min – max)	21	4 – 42	23	2 – 44	0.406

^a ^Fisher's exact test or Mann-Whitney U-test for group comparison

### Acceptability

The proportion of completed questionnaires was 100% (55/55) for hospital workers and 92.9% (39/42) for patients. The questionnaire was rated as easy or rather easy by 97.8% (87/89) of participants, and rather difficult or very difficult by 2.2% (2/89). The clinician rated scale filling as difficult or very difficult for 3.4% (3/88) of subjects. Median time to complete the questionnaire was 5 minutes (range 2 – 25 minutes). It was ≤ 10 minutes for 94.4% of subjects (N = 89).

### Score distributions

Median values were 40 and 36 for scores related to country of origin and host country, respectively. The latter relatively high value was in keeping with a length of stay in Switzerland exceeding 20 years for most participants. The observed variability for the country of origin score was 22 – 48. Score range for host country was 23 – 47. Thus, there was no floor effect for either score. A ceiling effect was observed for the country of origin subscale in only 2 participants (2.2%), whereas no such effect was present for the host country subscale.

### Internal consistency

The culture of origin and host culture scales achieved Cronbach's alphas of 0.77 and 0.73, respectively, when calculated for the whole sample (n = 93). Correlation coefficients between items and the corresponding total score (corrected for overlap) were ≥ 0.3 for 10 of the 12 items relative to country of origin, with lower consistency levels for items "importance to celebrate holidays" and "kindness and generosity of people". Correlation coefficients were ≥ 0.3 for 9 of 12 items relative to host country, with lower consistency observed for "enjoying TV programs or newspapers", "importance for children to have Swissfriends" and "kindness and generosity of Swiss people".

### Factor structure of the instrument

Principal component analysis of the 24 items of the complete scale identified a 4-factor structure that accounted for 46.8% of the total variance (Table 3). The first component was associated with 11 of the 12 items related to culture of origin (i.e. factor loadings ≥ 0.4), supporting their homogeneous membership in a single dimension. Eleven of the 12 items related to host culture contributed to the three other dimensions in a split-up manner, suggesting that the underlying construct might not be as homogeneous as for culture of origin. However, only 1 item related to host country and 3 items concerned with country of origin contributed to the first and higher order factors, respectively (loadings ≥ 0.4). This indicated little ambiguity with respect to item membership to dimensions related to culture of origin or host culture. The independence of the two 12-item subscales was further supported by the absence of a significant correlation between the two scores (Spearman r_S _= -0.042, P = 0.691).

**Table 3:** Principal component analysis with varimax rotation (n = 93)

	Items (English summary of French questions, I, S, P for Italian or Spanish or Portuguese respectively)	Factor 1	Factor 2	Factor 3	Factor 4
Country of origin	1. How much are (I, S, P) values part of your life?	0.44			-0.49
	2. How important is it to you to celebrate holidays in the (I, S, P) way ?				-0.63
	3. How important is it to you to raise your children with (I, S, P) values ?	0.68			
	4. How comfortable would you be in a group of (I, S, P) who don't speak French ?	0.61			
	5. How proud are you of being (I, S, P) ?	0.62			-0.36
	6. How much do you enjoy speaking (I, S, P) ?	0.61			
	7. How much do you enjoy TV programs or newspapers in (I, S, P) ?	0.51	-0.32		
	8. How much do you like to eat (I, S, P) food ?	0.62			
	9. Do you think (I, S, P) are kind and generous ?	0.43		-0.32	0.36
	10. How important would it be to you for your children to have (I, S, P) friends ?	0.66	-0.31		
	11. How comfortable do you feel to express your feelings in (I, S, P) ?	0.46			
	12. How important is it for you to think that you will rest, after your death, in (I, S, P) ?	0.42	-0.44		
Host country	13. How much are Swiss values part of your life ?			0.74	
	14. How important is it to you to celebrate holidays in the Swiss way ?			0.65	
	15. How important is it to you to raise your children with Swiss values ?			0.74	
	16. How comfortable would you be in a group of Swiss who don't speak (I, S, P) ?		0.71		
	17. How proud are you of a Swiss identity ?			0.75	
	18. How much do you enjoy speaking French ?		0.75		
	19. How much do you enjoy TV programs or newspapers in French ?		0.56		
	20. How much do you like to eat Swiss food ?		(0.29)	(0.29)	(0.28)
	21. Do you think Swiss people are kind and generous ?				0.76
	22. How important would it be to you for your children to have Swiss friends ?	0.46			
	23. How comfortable do you feel to express your feelings in French ?		0.77		
	24. How important is it for you to think that you will rest, after your death, in Switzerland ?		0.40	0.32	
*Explained variance*		*16.0%*	*12.1%*	*11.2%*	*7.5%*

Correlations between items and factors are reported if ≥ 0.30 (except for values in parentheses).

### Discriminant and convergent validity

Discriminant validity results are presented in Table 4. In keeping with the hypothesis, patients displayed significantly lower scores for host culture than hospital staff (median values 35 vs 37, P = 0.016). A similar trend was observed for culture of origin (median values 39 vs 41, P = 0.054). The culture of origin score was consistently and significantly higher than the host culture score, with a median difference of 4 points for both samples.

**Table 4:** Discriminant validity

	Hospital staff (n = 55)		Patients (n = 38)		P^a^
	Median	Min – Max	Median	Min – Max	
A. Score (country of origin)	41	24 – 48	39	22 – 48	0.054
B. Score (Switzerland)	37	23 – 47	35	26 – 43	0.016
Difference (A – B)	4	(-19) – 20	4	(-18) – 21	0.958
P^b^	<0.001		0.006		

^a ^Mann-Whitney U-test for group comparison

^b ^Wilcoxon signed ranks test for within-subject comparison

Convergent validity results are presented in Table 5. On the one hand, higher score for the Swiss culture was significantly associated with younger age at arrival (r_S _= -0.27, P = 0.008) and longer stay in host country (r_S _= 0.26, P = 0.011). On the other hand, lower score for the culture of origin was significantly associated with longer stay in Switzerland (r_S _= -0.31, P = 0.003), but not with younger age at arrival. More frequent return to country of origin was associated with higher score on the scale referring to country of origin (P = 0.001), whereas more frequent contact through phone calls or electronic mail did not show such an association.

**Table 5:** Convergent validity (n = 93)

	Score (country of origin)		Score (Switzerland)	
		P		P
Age at arrival in Switzerland (r_S _correlation)	0.13	0.209^a^	-0.27	0.008^a^
Length of stay in Switzerland, yrs (r_S _correlation)	-0.31	0.003^a^	0.26	0.011^a^
Frequency of returns to country of origin (median, min-max)				
More than once a year (n = 37)	42 (24 – 48)	0.001^b^	36 (23 – 43)	0.685^b^
Once a year (n = 39)	39 (29 – 48)		37 (26 – 44)	
Less than once a year (n = 17)	34 (22 – 44)		35 (28 – 47)	
Frequency of contacts with country of origin (median, min-max)		0.192^b^		0.676^b^
More than once a week (n = 28)	41 (24 – 48)		37 (24 – 43)	
Once a week (n = 25)	41 (32 – 46)		37 (23 – 41)	
Less than once a week (n = 40)	38 (22 – 48)		35 (26 – 47)	

^a ^Spearman correlation coefficient (r_S_)

^b ^Kruskal-Wallis one-way analysis of variance

## Discussion

The present study indicated that the questionnaire was well accepted and rapid (≤ 10 minutes for 94.4% of participants). The internal consistency of the scores referring to country of origin and host country was adequate (Cronbach's alpha ≥ 0.70) and comparable to values reported by Cortés et al. for their proposed 9-item scales for American and Puerto Rican cultures [13]. Principal component analysis addressed the underlying dimensionality of the instrument: it supported the concept of a two-dimensional model, with involvements in culture of origin and host culture independent from each other. Such a model is in keeping with the proposal by Cortés that each culture should be assessed independently. This two-dimensional model also contrasts with the older unidirectional model of assimilation or acculturation implying that involvement in a new culture occurs at the expense of the culture of origin. The identified factor structure also suggested that adherence to host culture may involve different components, whereas culture of origin appeared as a more homogeneous concept. Construct validity was approached by examining three sets of logical hypotheses. Firstly, scores for the Swiss culture were significantly higher among hospital workers than among patients treated for alcohol-related disorders. Secondly, involvement in host culture significantly increased, whereas scores for culture of origin decreased with increasing length of stay in Switzerland. Thirdly, more frequent journeys to country of origin were associated with maintaining higher involvement in the culture of origin.

The large observed interindividual variability of scores related to cultures of origin and host countries is in accordance with recent research emphasizing individual variations in the experience of biculturalism [14]. However, the two proposed subscales did not yet assess biculturality, i.e. the degree to which individuals are *simultaneously *immersed in both cultures. Thus, two hypotheses deserve to be examined in future studies. A first hypothesis is that bicultural identity, already known to be relevant in mental health, can be measured with a single instrument among migrants from different countries of origin. A second hypothesis is that a strong bicultural identity is associated with better mental health and treatment outcome, in contrast with low cultural involvement in both cultures or mono-culture, i.e. preferential adherence to either culture of origin or host culture. Similarly, several authors have postulated that individuals who have the ability to effectively alternate their use of culturally appropriate behavior may well exhibit higher cognitive functioning and mental health status than people who are monocultural [15].

It is noteworthy that, although directly adapted from instruments used in other cultural contexts, mainly North America, our scale seems to be valid among a sample of South European immigrants in Switzerland. It is reasonable to deduce that the process of acculturation in economical migrants might possess some transposable characteristics. In this perspective, the proposed scale could be valuably tested in other European settings (e.g. Turkish immigrants in Germany). However, there is no reason to believe that such a scale would measure relevant dimensions among precarious migrants (e.g. asylum seekers) for whom other short-term adaptation issues are at stake.

The present study has several limitations. Firstly, although we designed the scale taking into account existing instruments, it is not a translation of a previously validated scale. The proposed instrument rather adapted and complemented earlier ones on the basis of our clinical experience with immigrants in the Geneva community. Secondly, we used a pragmatic approach, pooling participants with comparable migration motives and patterns (i.e. Italian, Spanish and Portuguese subjects). Validation of the instrument with respect to its use in individuals from different cultures remains to be examined. Thirdly, patients and healthy subjects were not matched for socio-demographic characteristics and the influence of such factors on the observed differences cannot be ruled out. Fourthly, although no clinical assessment was performed, we considered hospital workers as "participants without a clinical diagnosis" and compared them as such with patients. This methodology has been used elsewhere for validating cross-cultural instruments [16].

Concerning the sample characteristics, the higher proportion of females among hospital workers may reflect the gender composition of the hospital staff at the time of the study (67% female, 33% male). It is also in keeping with the overrepresentation of males among alcohol dependent individuals who seek treatment. For various reasons, women with drinking problems are less likely than men to utilize alcohol-specific treatment services [17]. The difference with respect to country of origin may be related to the level of integration in Switzerland of the corresponding national communities. Portuguese migrated to Switzerland more recently than Italians and Spaniards, so that their likelihood to be employed as professionals in the public hospital was lower. As patients were recruited in an alcohol treatment facility, they all had alcohol-related disorders. This category of patients was chosen for convenience and thus the present study does not pretend to address cultural identity with respect to substance-related disorders. However, several studies conducted in diverse settings, countries and contexts have demonstrated that alcohol related behaviors are partly determined by socio-cultural and ethnic factors [18,19].

## Conclusion

The present study introduces a French language psychometric scale to be used by mental health professionals in the assessment of bicultural identity among economical migrants. Results provide elements of validation among Italian, Portuguese and Spanish immigrants living in Switzerland. Further studies are needed to address discriminant and predictive validity of the proposed instrument among larger populations of psychiatric patients, including more recent immigrants and different diagnostic categories.

## Competing interests

The author(s) declare that they have no competing interests.

## Authors' contributions

AE conceived the study and drafted the manuscript. NJP conducted the interviews and coordinated the study. MGF performed the statistical analysis. All three authors participated in the design of the study.

## Pre-publication history

The pre-publication history for this paper can be accessed here:


